# Local and systemic host responses to influenza and concurrent or sequential SARS-CoV-2 infection

**DOI:** 10.3389/fcimb.2026.1725731

**Published:** 2026-01-21

**Authors:** Tong Zhu, Liying Wang, Chaoying Yan

**Affiliations:** 1 Department of Neonatology and Institute of Pediatrics, Children’s Medical Center, First Hospital of Jilin University, Jilin University, Changchun, Jilin, China; 2 Department of Molecular Biology, College of Basic Medical Sciences, Jilin University, Changchun, Jilin, China

**Keywords:** infection, influenza, influenza virus, lung lesion, SARS-CoV-2, systemic response

## Abstract

Influenza is an acute respiratory infectious disease caused by the influenza virus, which has been circulating in humans for over a century. In contrast, COVID-19, caused by the novel SARS-CoV-2, emerged recently in December 2019. Following nearly four years of pandemic, the acute phase of SARS-CoV-2 has transitioned towards an endemic state, suggesting a trend of long-term coexistence with humans. Concurrent or sequential coinfection with influenza and SARS-CoV-2 has been clinically observed to exacerbate pulmonary pathology and systemic inflammation in affected individuals. This review discusses the impact and elucidates the potential underlying mechanisms by which influenza and SARS-CoV-2 coinfection aggravates local lung injury and systemic host responses, aiming to inform improved prevention and clinical management strategies.

## 1 Introduction

Influenza is an acute respiratory infectious disease caused by the influenza virus, characterized by high transmissibility and seasonality, primarily driven by influenza A (IAV) and B (IBV) viruses. Clinical manifestations range from systemic symptoms (e.g., high fever, fatigue) and respiratory signs (e.g., sore throat, cough) to severe outcomes including acute respiratory distress syndrome (ARDS) and death. The 1918 influenza pandemic alone claimed over 50 million lives (Johnson and Mueller, 2002). Since then, influenza has persisted through periodic pandemics, recurrent epidemics, and annual sporadic cases, remaining a significant global health threat. Current estimates attribute approximately 500,000 annual deaths to influenza (Javanian et al., 2021).

The public health landscape of influenza has been further complicated by the emergence of SARS-CoV-2, the causative agent of COVID-19, which first appeared in December 2019 and rapidly escalated into a global pandemic. As of September 2024, there have been around 776 million confirmed cases of COVID-19, resulting in over 7 million reported deaths (World Health Organization, 2024). While the virulence of circulating SARS-CoV-2 variants may have attenuated over time, leading to a transition towards endemicity and long-term coexistence with humans, the risk of co-circulation with influenza viruses persists. Consequently, the probability of influenza and SARS-CoV-2 coinfection, either concurrently or sequentially, is significantly heightened, particularly during seasonal influenza periods. This review will summarize and discuss current understanding of the local and systemic host immune responses elicited by influenza and SARS-CoV-2 coinfection.

## 2 Comparative virology of influenza virus and SARS-CoV-2

Influenza virus and SARS-CoV-2 are both major pathogens responsible for acute respiratory infections in humans. This section delineates the key virological similarities and differences between them.

### 2.1 Shared virological characteristics

Influenza viruses and SARS-CoV-2 share several fundamental virological characteristics. Firstly, both are enveloped RNA viruses, albeit with differing genomic polarities: influenza virus possesses a single-stranded negative-sense RNA (ssRNA^-^) genome, whereas SARS-CoV-2 has a single-stranded positive-sense RNA (ssRNA^+^) genome. Secondly, the viral envelopes of both, derived from the host cell membrane, are studded with distinct glycoproteins crucial for infectivity. The influenza envelope is predominantly equipped with hemagglutinin (HA), neuraminidase (NA), and the M2 ion channel, while the SARS-CoV-2 envelope contains the spike (S), envelope (E), and membrane (M) proteins. A third commonality lies in their mechanism of cellular entry. Both pathogens initiate infection by utilizing specific viral envelope glycoproteins to bind to host cell surface receptors: influenza HA binds to sialic acids (Samji, 2009), and the SARS-CoV-2 S protein engages angiotensin-converting enzyme 2 (ACE2) (Shang et al., 2020). Finally, their tropism overlaps significantly, as both primarily target epithelial cells throughout the respiratory tract, leading to comparable routes and modes of transmission (Gagliardi et al., 2022; Luo et al., 2023).

### 2.2 Distinct virological features

Despite these similarities, influenza viruses and SARS-CoV-2 exhibit pronounced differences in their genomic architecture, replication mechanisms, and evolutionary dynamics. A primary distinction lies in their genomic organization. The influenza A and B virus genomes are segmented, comprising eight single-stranded RNA segments totaling approximately 13,600 nucleotides, with each segment (890-2,340 nucleotides) encoding one to two proteins (Lee et al., 2017). In stark contrast, the SARS-CoV-2 genome is a non-segmented, single RNA strand of about 29,800 nucleotides, containing 13 open reading frames (ORFs) that encode at least 14 proteins (Ahmed et al., 2020; Kawasaki et al., 2023). This fundamental disparity in genome structure is a postulated contributor to their divergent replication kinetics; influenza viruses typically replicate more rapidly than SARS-CoV-2 (Pinky and Dobrovolny, 2020; Zarkoob et al., 2022), potentially owing to the replication efficiency of shorter, segmented RNA molecules. Secondly, the mechanisms of viral entry and release differ substantially. Influenza virus attachment via HA to sialic acid is followed by clathrin-mediated endocytosis (Sun and Whittaker, 2013). Subsequent viral egress requires neuraminidase (NA) activity to cleave sialic acid receptors, facilitating the release of new virions from the host cell membrane (Shtyrya et al., 2009). Conversely, SARS-CoV-2 attachment to ACE2 via its S protein permits direct, TMPRSS2-mediated fusion at the plasma membrane, bypassing the endosomal pathway for efficient entry (Jackson et al., 2022). A third critical difference is their inherent mutation rate, which is significantly higher in influenza viruses than in SARS-CoV-2 (Kawasaki et al., 2023). This can be attributed to two key factors: 1) Replication Fidelity: The influenza ssRNA^-^ genome must be transcribed into positive-sense RNA by the viral RNA-dependent RNA polymerase (RdRp), which lacks proofreading. Errors during this process readily introduce mutations that are incorporated into viral proteins (antigenic drift). The SARS-CoV-2 ssRNA^+^ genome, while also replicated by an error-prone RdRp, can directly serve as mRNA for translation, potentially offering a slightly more constrained window for initial mutational incorporation. 2) Genome Segmentation: The segmented nature of the influenza genome enables antigenic shift (reassortment), a form of large-scale genetic exchange. When two distinct influenza viruses co-infect a single cell, progeny virions can incorporate mixed sets of genomic segments, potentially giving rise to novel pandemic strains, a phenomenon not possible with the non-segmented SARS-CoV-2 genome.

## 3 Exacerbation of pulmonary pathology in influenza and SARS-CoV-2 coinfection and underlying mechanisms

The phenomenon of influenza and SARS-CoV-2 coinfection emerged concurrently with the COVID-19 pandemic in late 2019. Initial reports primarily documented influenza virus coinfection in patients already diagnosed with COVID-19. Notably, approximately 50% of patients hospitalized in the early stage of the pandemic were coinfected with influenza viruses, predominantly influenza A virus (IAV) (Ma et al., 2020; Cheng et al., 2021). However, these early reports represent the upper limit of the reported coinfection rate. As the pandemic evolved, factors including SARS-CoV-2 mutation towards reduced virulence and widespread public health interventions (e.g., mask-wearing) collectively decreased the likelihood of influenza coinfection in COVID-19 patients (Olsen et al., 2021). Surveillance data from various countries and regions indicate that the rate of influenza coinfection in COVID-19 patients has since significantly declined to a range of 4% to 10% (Swets et al., 2022; Morales-Jadán et al., 2023; Milano et al., 2024). A recent meta-analysis covering the period from the pandemic onset to July 2024 reported an overall pooled coinfection rate of 14% (Golpour et al., 2025)*,* although this incidence remains higher than that of other respiratory viral coinfections. Presently, while the peak of the SARS-CoV-2 pandemic has passed, seasonal influenza epidemics persist. The recent resurgence of influenza incidence, even surpassing levels observed during the height of the COVID-19 pandemic, has refocused attention on the clinical significance of influenza and SARS-CoV-2 coinfection.

### 3.1 Influenza and SARS-CoV-2 coinfection exacerbates pulmonary pathology, with severity modulated by infection timing and sequence

Influenza combined with SARS-CoV-2 infection usually worsens the lung lesions in infected individuals. Clinical and experimental evidence indicates that the severity of the disease is significantly influenced by the sequence and interval between the two viral infections. Much of the initial evidence derives from models where influenza virus infection precedes SARS-CoV-2. After infecting ferrets or K18-hACE mice with IAV and giving SARS-CoV-2 at 5 days later, it was found that the ferret’s alveolar interstitial inflammatory cell infiltration and fibrin exudation in the alveolar cavity were significantly increased, and their necrotizing pneumonia was aggravated. Alveolar interstitial inflammation was more severe in mice than in ferrets, and was accompanied by necrosis of bronchial epithelial cells and hemorrhage in the alveolar cavity. The death of these mice accelerated (Bao et al., 2021). Other researchers found that the lung injury of mice infected with non-lethal IAV (A/X31) followed by SARS-CoV-2 infection 3 days later was more severe and long lasting (Clark et al., 2024). This is similar to the situation observed in patients coinfected with influenza virus and SARS-CoV-2. A Meta-analysis of 59 studies involving 16,643 patients infected with SARS-CoV-2 showed that the patients coinfected with influenza virus were more likely to have dyspnea and increased mortality (Krumbein et al., 2023). However, the timing of influenza coinfection with SARS-CoV-2 directly affects the severity of the illness. It was found that mice infected with SARS-CoV-2 on the 2nd and 5th day of IAV infection would aggravate their respiratory symptoms and significantly increase their mortality. But mice infected with SARS-CoV-2 on the 8th day of IAV infection did not worsen their respiratory symptoms (Achdout et al., 2021). A similar phenomenon has been found in experiments with hamsters. After infection with SARS-CoV-2 at 3 h of H1N1 infection, the hamster’s lung symptoms remained severe, but after infection with SARS-CoV-2 at 48 h of H1N1 infection, the hamster’s lung symptoms were significantly reduced (Di Pietro et al., 2024). Importantly, the exacerbated pathology is not confined to the scenario where influenza is the primary infection. Studies in K18-hACE2 mice have demonstrated that prior infection with SARS-CoV-2 also exacerbates subsequent H1N1 infection, leading to more pronounced weight loss, enhanced viral shedding, and more extensive lung consolidation, with a shorter interval (7 days) between infections proving more detrimental than a longer one (14 days) (Li et al., 2021). Furthermore, Kim (Kim et al., 2022) demonstrated that in K18-hACE2 mice, coinfection with IAV (H1N1) and SARS-CoV-2 with a short 3 day interval, regardless of the infection order, led to increased mortality, greater weight loss, higher pulmonary viral loads, and enhanced immune cell infiltration compared to single infections.

### 3.2 Potential mechanisms underlying aggravated lung pathology following sequential infection

**Figure 1:**
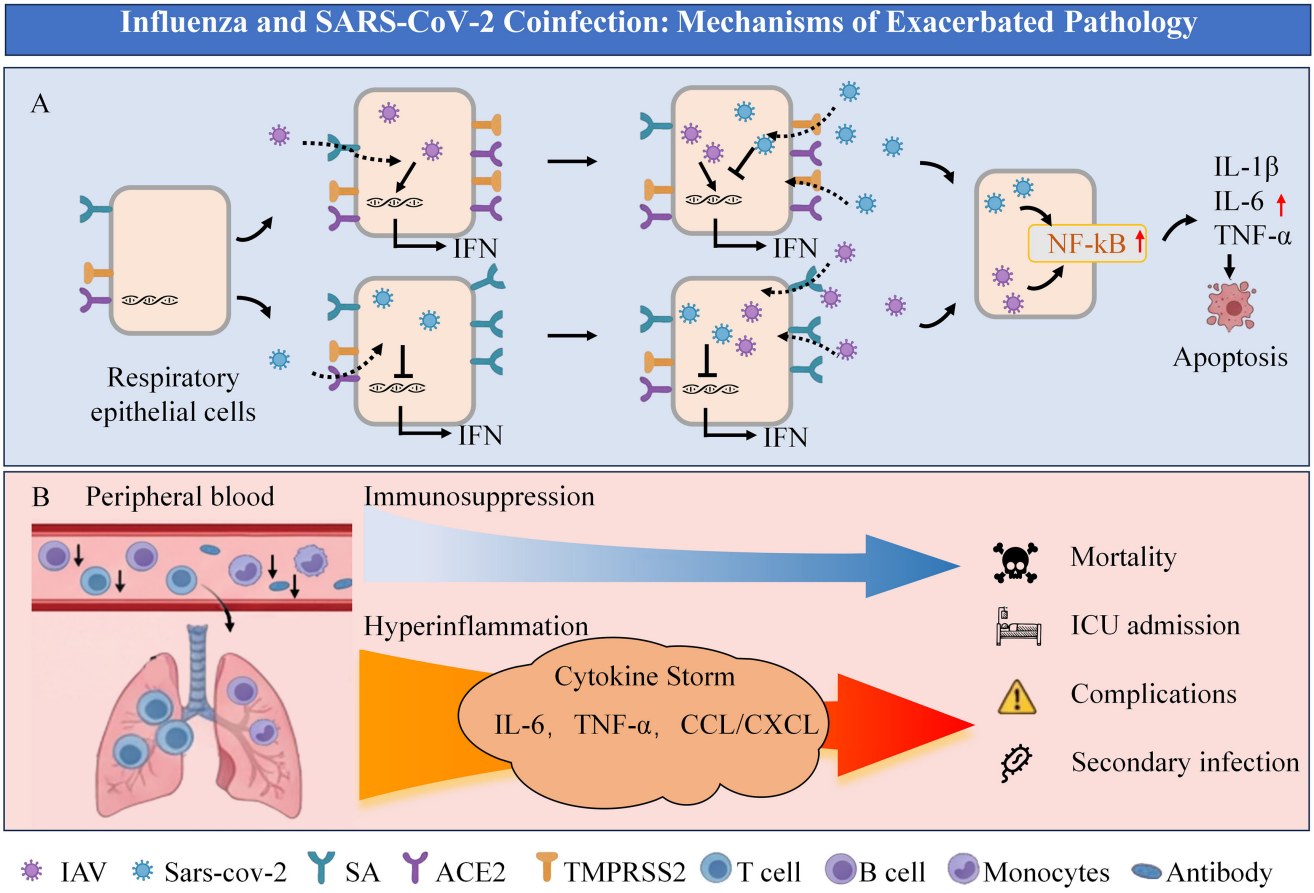
Influenza and SARS-CoV-2 coinfection: mechanisms of exacerbated pathology. **(A)** Alterations in respiratory epithelial cells during sequential infection with influenza and SARS-CoV-2. **(B)** Systemic immune response & outcomes.

The exacerbation of lung injury in influenza and SARS-CoV-2 coinfection may be attributed to several interconnected mechanisms. It is important to distinguish between sequential infection (where one virus precedes the other) and concurrent coinfection, as the underlying mechanisms can differ based on the order of viral exposure (**Figure 1A**).

#### 3.2.1 Mechanisms when influenza infection precedes SARS-CoV-2

(1) Upregulation of Host Entry Receptors for SARS-CoV-2. Influenza virus infection can upregulate the expression of ACE2 and TMPRSS2 in respiratory epithelial cells (Bai et al., 2021; Kim et al., 2023). *Notably, ACE2 is an interferon-stimulated gene (ISG)* (Ziegler et al., 2020), and its upregulation is a common host response observed during various respiratory viral infections beyond IAV, such as rhinovirus and cytomegalovirus (Chang et al., 2020*;*Blume et al., 2021*;*Perera et al., 2023). However, despite this shared mechanism, the IAV/SARS-CoV-2 pairing is of primary clinical concern due to its higher coinfection frequency and more severe outcomes. This receptor upregulation is particularly significant in the lower respiratory tract, where baseline ACE2 expression is relatively low (Hou et al., 2020; Liu et al., 2020; Ortiz et al., 2020). This virus-mediated increase in receptor availability may facilitate SARS-CoV-2 infection even at lower viral inoculums. Supporting evidence comes from human pluripotent stem cell-induced alveolar type II organoids (hiAT2), where ACE2 and TMPRSS2 mRNA levels were elevated at 12 and 48 h post-IAV infection. Consistent upregulation of these factors was also confirmed in A549, Calu-3, and NHBE cell lines, as well as in lung tissues of IAV-infected K18-hACE2 mice (Bai et al., 2021). Crucially, IAV infection has been shown to enhance ACE2 expression specifically in the murine distal respiratory tract (alveoli), thereby potentially enabling more extensive SARS-CoV-2 invasion and subsequent acute lung injury (Schweitzer et al., 2021).

(2) Suppression of Antiviral Interferon Response by SARS-CoV-2. SARS-CoV-2 employs multiple strategies to antagonize the type I interferon (IFN-I) pathway, a critical component of innate antiviral defense. The viral non-structural protein Nsp1 can inhibit the binding of interferon regulatory factor 3 (IRF3) to the IFN-β promoter, thereby suppressing IFN-β production (Kumar et al., 2021). Additionally, the ORF6 protein impedes the nuclear translocation of STAT1 and STAT2, effectively blocking IFN signaling and the expression of ISGs (Miorin et al., 2020). While influenza virus is a potent inducer of IFN-I (Feng et al., 2021), concurrent or shortly sequential infection with SARS-CoV-2 may create a temporal window wherein SARS-CoV-2 proteins inhibit the initiation or efficacy of the IFN-I response elicited by influenza, leading to uncontrolled viral replication. The success of SARS-CoV-2 in establishing infection under these conditions highlights its potent ability to break through a pre-activated antiviral state. Furthermore, the inherently slower replication kinetics of SARS-CoV-2 compared to influenza virus (Jin et al., 2024) may render it more susceptible to suppression by a robust, pre-established IFN response, which could explain the mitigated severity observed with longer intervals between infections (Achdout et al., 2021; Di Pietro et al., 2024).

#### 3.2.2 Mechanisms when SARS-CoV-2 infection precedes influenza

Conversely, a different set of mechanisms comes into play when SARS-CoV-2 infection occurs first.

(1) Enhanced Susceptibility to Influenza Virus via Sialic Acid Modulation. Human influenza viruses primarily utilize α-2,6-linked sialic acids for cellular entry, with some strains also recognizing α-2,3-linked sialic acids (Abdelrahman et al., 2020). Intriguingly, infection with SARS-CoV-2 was found to significantly increase the abundance of α-2, 3-linked sialic acids on the surface of hiAT2 organoids (Kim et al., 2023). This virus-induced alteration in the host glycan landscape could potentially facilitate increased adhesion and entry of influenza viruses, thereby promoting further infection and spread within the respiratory tract. This mechanism may represent a novel viral synergy contributing to the heightened lung pathology observed in coinfected individuals.

(2) Permissive Environment from Delayed Innate Immunity. SARS-CoV-2 is known for its ability to delay and suppress the early IFN-I response (Blanco-Melo et al., 2020; Gilbert-Girard et al., 2024). When SARS-CoV-2 infects first, it creates an initial state of low antiviral alert. The subsequent introduction of the fast-replicating influenza virus into this ‘immunologically permissive’ environment can lead to its unchecked initial replication. The combination of SARS-CoV-2-mediated IFN suppression and the rapid replication kinetics of influenza virus creates a synergistic effect, allowing for a swift and high viral load buildup that drives severe pathology.

#### 3.2.3 Shared mechanisms across sequences

Beyond sequence-specific mechanisms, a key driver of severe lung pathology in coinfection, regardless of the order of viral exposure, is the synergistic activation of inflammatory pathways. A study in human lung epithelial cells demonstrated that the SARS-CoV-2 S protein and the IAV HA protein can synergistically activate the NF-κB pathway. This co-stimulation resulted in approximately twice the activation strength of the pathway and a 6–9 fold increase in the expression/secretion of pro-inflammatory cytokines like IL-1β and IL-6 compared to stimulation by either viral protein alone. This massively amplified inflammatory response led to significantly increased epithelial cell apoptosis, disrupted vascular repair capacity, and excessive immune cell recruitment, culminating in aggravated local tissue damage (Anggraeni et al., 2024). This molecular synergy provides a fundamental explanation for the intensified ‘cytokine storm’ observed in coinfected hosts. This time-dependent synergistic effect on inflammation, whereby two distinct viruses activate a common pathway (e.g., NF-κB) through different receptors, leading to excessive immune activation, requires the near-simultaneous presence of both viral stimuli and thereby explains the critical narrow time window for severe pathology. Additionally, the requirement for a narrow time window is further explained by the maturation kinetics of the early antiviral state, which is primarily mediated by IFN-I. As demonstrated in a human airway epithelial model, the IFN-β response to IAV infection peaks at 72 h post-infection (h p.i.) for A/H3N2 and at 120 h p.i. for A/H1N1 strains (Gilbert-Girard et al., 2024). Therefore, a short interval allows a second virus to invade before this potent IFN-I response has peaked, enabling immune escape and severe coinfection. Conversely, a longer interval permits the establishment of a mature antiviral state that effectively suppresses the second virus, thereby mitigating disease severity.

## 4 Systemic host response to influenza and subsequent or concurrent SARS-CoV-2 infection

In individuals coinfected with influenza and SARS-CoV-2, severe systemic manifestations often accompany the pronounced local respiratory symptoms. A study of 505 patients with influenza virus and SARS-CoV-2 coinfection demonstrated a significantly elevated risk of acute kidney injury, acute heart failure, secondary bacterial infection, intensive care unit (ICU) admission, and death compared to infection with a single virus (Zheng et al., 2021). Data from the United Kingdom indicated that coinfection with influenza virus and SARS-CoV-2 was associated with a 5.92-fold increase in mortality compared to single virus infection (Stowe et al., 2021). Consistent with these clinical observations, animal studies have shown that ferrets and mice coinfected with influenza virus and SARS-CoV-2 exhibit higher fever, greater weight loss, prolonged duration of clinical symptoms, and increased mortality (Bao et al., 2021). Furthermore, studies in mice have revealed that influenza virus infection followed by SARS-CoV-2 exacerbates extrapulmonary manifestations, including SARS-CoV-2-associated encephalitis (Clark et al., 2024).

Coinfection with influenza virus and SARS-CoV-2 leads to significant abnormalities in host immune cells and responses. Research has shown that absolute peripheral T cell counts are reduced in patients with COVID-19, particularly in severe cases (Chen et al., 2020; Popescu et al., 2022). A similar reduction is observed in influenza patients (Putter and Seghatchian, 2023), suggesting that coinfection with both viruses may cause a more profound depletion of T cells. It is critical to interpret this systemic lymphopenia from two potential, non-mutually-exclusive perspectives: it may not merely reflect immunosuppression, but also signify a massive recruitment of immune cells to the site of infection, which could conversely lead to uncontrolled local immune activation and immunopathology (**Figure 1B**). On one hand, the recruited immune cells can drive localized and systemic hyperinflammation. Moreover, coinfected individuals experience a more intense inflammatory cytokine storm. Notably, a study in mice coinfected with IAV and SARS-CoV-2 demonstrated a concurrent significant reduction of T cells, B cells, and monocytes in the periphery, alongside a marked increase in these immune populations in the bronchoalveolar lavage fluid (BALF). This localized accumulation was accompanied by significantly elevated levels of pro-inflammatory cytokines (e.g., TNF-α, IL-1α, IL-6) and chemokines (e.g., CCL3, CCL4, CCL5, CXCL9) in the BALF compared to single infections (Kim et al., 2022). A study in hamsters also reported that coinfection with influenza and SARS-CoV-2 significantly increased systemic IL-6 levels (Kinoshita et al., 2021). These findings provide direct experimental evidence for the exacerbated inflammatory response postulated above, which is driven by the recruited immune cells and leads to severe tissue damage. On the other hand, a sustained and profound viral challenge can lead to genuine T cell exhaustion and systemic immunosuppression. This is supported by animal experiments; mice exposed to H1N1 influenza A virus (IAV) during the recovery phase from SARS-CoV-2 infection exhibited reductions in various lymphocyte populations. These included peripheral CD3^+^ T cells and T regulatory cells (Tregs), as well as pulmonary CD3^+^ T cells, T helper 1 (Th1), Th2, and Th17 cells, contributing to more severe disease (Li et al., 2021). Additionally, influenza and SARS-CoV-2 coinfection can alter other immune parameters. It was found that coinfection significantly reduced the numbers of monocytes and B cells, as well as the levels of serum total IgG, viral neutralizing antibody titers, and the virus-specific IFN-γ^+^CD4^+^ T cell response (Kim et al., 2022). This state of immune dysregulation and functional impairment in coinfected individuals also predisposes them to secondary bacterial infections, which are a major contributor to severe disease in both influenza and SARS-CoV-2 monoinfections (Rice et al., 2012; Smith et al., 2022; Goncheva et al., 2023).

## 5 Conclusion

The seasonal prevalence of influenza viruses, coupled with the established endemic presence of SARS-CoV-2, creates ample opportunities for their co-circulation and coinfection. As both viruses are primarily transmitted via the respiratory route and can mutually enhance the expression of their respective cellular receptors upon infection, the resultant impact on the pulmonary and systemic immune systems typically leads to an aggravated disease presentation in coinfected individuals. It is critical to emphasize that disease severity is determined not only by the direct cytopathic effects of the viruses but also by the nature of the host’s immune response. An overly robust immune reaction, particularly in the early stages of infection, can itself be a major driver of severe pathology. Furthermore, the temporal sequence of viral exposure is a decisive factor influencing both local and systemic outcomes; an inappropriate therapeutic intervention timed without considering this interplay may exacerbate the condition. For instance, administering exogenous interferon during the peak of an influenza-induced endogenous IFN response could potentially precipitate an excessive inflammatory reaction, thereby worsening cytokine storm-mediated tissue damage (Gu et al., 2021). When considering coinfection in the elderly, it is important to acknowledge the possibility of coincidental coinfection. However, clinical evidence moves beyond coincidence, demonstrating that coinfected individuals are significantly older than those with monoinfections and bear a disproportionately higher burden of comorbidities. Consequently, these patients face substantially elevated risks of acute kidney injury, acute heart failure, and mortality (Zheng et al., 2021). This vulnerability in the elderly is driven by immunosenescence, which impairs viral clearance, thereby creating a permissive environment for enhanced viral replication and systemic cytokine storms (Mai et al., 2025). In summary, as the acute phase of the COVID-19 pandemic recedes but seasonal influenza epidemics persist, maintaining a high alert for influenza and SARS-CoV-2 coinfection is imperative. Heightened individual protective measures during influenza seasons are essential to prevent coinfection and mitigate the risk of severe illness.
